# Supplementary material for: Curated and harmonised transcriptomics datasets of interstitial lung diseases
Source: Data Brief. 2025 Oct 14;63:112139. doi: 10.1016/j.dib.2025.112139 (PMC12581653; doi:10.1016/j.dib.2025.112139)
Supplement: Supplementary file 1 [file mmc1.zip › Supplementary_material/RNA-seq/fastQC reports/GSE124685/SRR8397181_fastqc.html]

SRR8397181.fastq.gz FastQC Report 

FastQC Report

Tue 30 Jan 2024  
SRR8397181.fastq.gz

## Summary

- Basic Statistics
- Per base sequence quality
- Per sequence quality scores
- Per base sequence content
- Per sequence GC content
- Per base N content
- Sequence Length Distribution
- Sequence Duplication Levels
- Overrepresented sequences
- Adapter Content

## Basic Statistics

| Measure | Value |
| --- | --- |
| Filename | SRR8397181.fastq.gz |
| File type | Conventional base calls |
| Encoding | Sanger / Illumina 1.9 |
| Total Sequences | 20275834 |
| Sequences flagged as poor quality | 0 |
| Sequence length | 8-369 |
| %GC | 54 |

## Per base sequence quality

## Per sequence quality scores

## Per base sequence content

## Per sequence GC content

## Per base N content

## Sequence Length Distribution

## Sequence Duplication Levels

## Overrepresented sequences

| Sequence | Count | Percentage | Possible Source |
| --- | --- | --- | --- |
| GGGCCACGAGCTGAGTGCGTCCTGTCACTCCACTCCCATGTCCCTTGGGA | 328130 | 1.6183304716343605 | No Hit |
| AAGCTCAGGGAGAGCCCTGTTAGGGCCGCCTCTGGCCCTAGTCTCAGACC | 316967 | 1.5632747831729144 | No Hit |
| GGGAATGTCTACGTGCGTATGCACGTGGCACTCTCTGCCCGAGGTCCGGG | 261034 | 1.2874143672709097 | No Hit |
| CCACCTTCTCCATCGGCTCCACTGGCCTCGTGGTGTATGACTACCAGCAG | 240841 | 1.1878229028704812 | No Hit |
| ATGATGTAGCAGCAGGTGCCAGGGGCTGGCTTGTAGGCGATCAGCAGCTG | 189175 | 0.9330072439930214 | No Hit |
| ACACTGAGGACTCTGTTCCTCCCCTTTCCGCCTAGGGGAAAGTCCCCGGA | 143455 | 0.7075171359165794 | No Hit |
| AAAAGTGATGGACTGCCCATTGCTGGAGAAGACCTTCTCTCCTACTGTCA | 119826 | 0.5909793895531005 | No Hit |
| ATCAAATCCTGCAGACAAGGGGAGCCCTCAGTCTGCAGGGCTCCATAATG | 94066 | 0.46393159462639116 | No Hit |
| TCTCTGGGCTTCTATTTCGACCGCGATGATGTGGCTCTGGAAGGCGTGAG | 76997 | 0.3797476345485961 | No Hit |
| TGGTTCCTGGCAAGCCCATGTGTGTTGAGAGCTTCTCAGACTATCCACCT | 71490 | 0.35258722279931864 | No Hit |
| GGCTGCCTGGAGCCCCTGGTGTCCCTGGAGAGCGTGGAGAGAAGGGGGAG | 68562 | 0.3381463864815622 | No Hit |
| AGCGGTTCCGCTGCCCTGAGGCACTCTTCCAGCCTTCCTTCCTGGGCATG | 68183 | 0.3362771662068253 | No Hit |
| TCACACTTCATGATGGAGTTGAAGGTAGTTTCGTGGATGCCACAGGACTC | 61953 | 0.3055509331946592 | No Hit |
| ACACTGAGGACTCTGTTCCTCCCCTTTCCGCCTAGGGAAAGTCCCCGGAC | 59509 | 0.29349717501139533 | No Hit |
| ACACCCACCGCAACTGTCTGTCTCATATCACGAACAGCAAAGCGACCCAA | 58539 | 0.28871315478317683 | No Hit |
| GAGCTCCTCATCTAGATGAGCTGGAAGCCCTGGAGGGCCTCTCTCGCCAG | 56252 | 0.27743371739973804 | No Hit |
| GCTGCTACAGGAGAATAGCAGACAGGTATAGTTAAGAAACAACTTTATTT | 53659 | 0.26464509425358285 | No Hit |
| ACGCTCGTAGCCCTCGCGCTTCTCCTCGGCCAGTTCGCGGAAGAAGTGGC | 51306 | 0.2530401462154405 | No Hit |
| GGGTACTTCAGGGTCAGGATGCCACGCTTGCTCTGGGCCTCGTCGCCCAC | 49344 | 0.2433636022074357 | No Hit |
| CTAAGTGCTGTGTTGTCGTTCCCCCTGCTTAAAATAAAGTTGTTTCTTAA | 45631 | 0.22505116189055402 | No Hit |
| AACCCGCGTGCAACCTGTCCCGACTCTAGCCGCCTCTTCAGCTCGCCATG | 36855 | 0.18176810877421862 | No Hit |
| AGCGGTTCCGGTGTCCGGAGGCGCTGTTCCAGCCTTCCTTCCTGGGTATG | 35869 | 0.1769051768721326 | No Hit |
| TCGGGCGCCCCAGACACCAGGCGTCATGGTGGGCATGGGCCAGAAGGACT | 35485 | 0.17501129669931212 | No Hit |
| TCTTTCTGGCCTGGAGGCTATCCAGCGTACTCCAAAGATTCAGGTTTACT | 34411 | 0.16971435059095472 | No Hit |
| TCTGTCTTCTTCAGTTTCGACTTATCGAATTTCTCGATCTCAGCCATATC | 32237 | 0.15899222690420525 | No Hit |
| TCACACTTCATGATGGAGTTGAAGGTGGTCTCGTGGATGCCGCAAGATTC | 31894 | 0.1573005578956703 | No Hit |
| AAAAGTGATGGACTGCCCATTGCTAGAGAAGACCTTCTCTCCTACTGTCA | 31306 | 0.15440055388103888 | No Hit |
| ACACTGAGGACTCTGTTCCTCCCCTTCCGCCTAGGGAAAGTCCCCGGACC | 29833 | 0.1471357479056102 | No Hit |
| TGAAGTCCAGCGGCCTCTTCCCCTTCCTGGTGCTGCTTGCCCTGGGAACT | 28301 | 0.13957995513279503 | No Hit |
| GTCCGACGCACCTGTTTGCAGGCTGCCTGGCGCTCCTTGGTCTCGTTGTC | 27192 | 0.13411038973785244 | No Hit |
| TGAATCTTGAACTGAGTTCCACTTGTAAACTTCTTGTTTCTTGTGGTTCC | 26299 | 0.12970613194012143 | No Hit |
| GACAGACTCCAGCTTTGAAGGACTTTCCAGAGCCTTCCACAGCCCAAGGT | 26167 | 0.12905511063071437 | No Hit |
| CACCCTTGATTCCACCAATAGTCAGCTTGTCCTCGCTCAGGATTCCATCT | 25928 | 0.1278763675023183 | No Hit |
| TTGGCTTTCTCTTTCCTCTTCTCCTCCAGGGTGGCTGTCACTGCCTGGTA | 24985 | 0.12322551072375124 | No Hit |
| ACAGTGTATCTAGCTTTGGAAAACACAGGGGTCTGCCCTGTGAGCTGCTC | 24221 | 0.11945747829657709 | No Hit |
| AAAGCCAAGAAAACCAACGATGCCAGATACTGAATTTCTATAGCACTGAC | 23725 | 0.11701121640668394 | No Hit |
| TGTCCATGTCTTACTACTTTGACCGCGATGATGTGGCTTTGAAGAACTTT | 23489 | 0.11584726921713799 | No Hit |
| TGAGGCGGAGCACCAGAGAGCCTACCTGGAAGACACATGCGTGGAGTGGC | 23331 | 0.11506801643769622 | No Hit |
| CGGTTGTACTTGCGGATGTAGTGCAGATAGTCTCGGCGGATGACAATGGT | 23229 | 0.11456495451679077 | No Hit |
| ACCTACGGCGAGGGCGAGAGCGGCCCCATGGGCAACATCATGATCGATCC | 23215 | 0.1144959068021567 | No Hit |
| ACATAGCAATTCAGGAAATTTGACTTTCCATTCTCTGCTGGATGACGTGA | 22516 | 0.11104845305006936 | No Hit |
| AAGGCCCACCCGTGGAGGCCAGACCCAAAGCCCACGGTACCGAGGACAGG | 22450 | 0.11072294239536584 | No Hit |
| AATCGTGGTCAGCGTCATAGGTGGTAAAGGGCCTCCCACTGTGGAGGCTC | 22396 | 0.11045661549606296 | No Hit |
| TGCACTTCATCCTCATGAAGAAGTCCCGCTCTGTGGACATGTCTTTGCTT | 22170 | 0.10934198810268422 | No Hit |
| AACATCTGTGTTGGGGAGAGTGGAGACAGACTGACGCGAGCAGCCAAGGT | 22154 | 0.10926307642881669 | No Hit |
| CAGCTGGAGAAGGACTTCAGCAGCATGAAGAAGTACTGCCAAGTCATCCG | 22068 | 0.10883892618177876 | No Hit |
| TTTACACCTGCTAGGGTGTTCAAAGGTCAGTGCTATAGAAATTCAGTATC | 22025 | 0.1086268510582598 | No Hit |
| GGCCTTCTTCTGGCGCAGAGGAAGCAGGCGCATCTGGGTGTGGGCAATGA | 21655 | 0.10680201860007338 | No Hit |
| TGTTCCCTCTCCTCATGAGATTGGTGAAGAAAGTATTTGGCAAAGTTCTT | 21631 | 0.1066836510892721 | No Hit |
| TACTCGTGCGCCTCGCTTCGCTTTTCCTCCGCAACCATGTCTGACAAACC | 21086 | 0.10399572219815965 | No Hit |
| GCCAAGCACAAAGAGCTTGCTCCCTACGATGAGAACTGGTTCTACACGCG | 20948 | 0.10331510901105227 | No Hit |
| ATCACCGCCCTCGAGGCCAAGATTGCACAGCTGGAGGAGCAGCTGGACAA | 20509 | 0.10114996995931216 | No Hit |

## Adapter Content

Produced by FastQC (version 0.11.7)
